# Advanced cellulose-based hydrogel TiO_2_ catalyst composites for efficient photocatalytic degradation of organic dye methylene blue

**DOI:** 10.1038/s41598-024-61724-w

**Published:** 2024-05-13

**Authors:** Bang Cong Nguyen, Thu Minh Truong, Ngoc Thi Nguyen, Duong Ngoc Dinh, Dirk Hollmann, Mai Ngoc Nguyen

**Affiliations:** 1 School of Chemistry and Life Sciences, Hanoi University of Science and Technology, No. 1 Dai Co Viet Street, 10000 Hanoi, Vietnam; 2 Department of Chemistry, University of Rostock, Albert-Einstein-Straße 3A, 18059 Rostock, Germany; 3 Department Life, Light & Matter, Faculty for Interdisciplinary Research, University of Rostock, Albert-Einstein-Straße 25, 18059 Rostock, Germany

**Keywords:** Cellulose hydrogel, Immobilization, Microcrystalline cellulose, Tetrabutylphosphonium hydroxide, Titanium dioxide, Gels and hydrogels, Sustainability

## Abstract

Sustainable cellulose-based hydrogels are used in medicine and environmental science. Hydrogels’ porosity makes them excellent adsorbents and stable substrates for immobilizing photocatalysts to remove organic dyes. Despite their potential, the implementation of hydrogels for this purpose is still limited due to their high synthesis temperature and low cellulose content. To overcome these challenges, this study develops cellulose-based hydrogels, which have a high cellulose content and can be easily synthesized under ambient conditions. Containing a higher cellulose concentration than previous hydrogels, the synthesized hydrogels are more stable and can be reused numerous times in treatment operations. The hydrogel properties were investigated using Fourier transform infrared spectroscopy, X-ray diffraction and thermal analysis. Scanning electronic microscopy revealed that TiO_2_ nanoparticles were homogeneously distributed throughout the hydrogel's matrices. In addition, transparent hydrogels allow light to pass through, making them suitable substrates to remove organic dye. The results showed that the hydrogel with TiO_2_ was able to degrade nearly 90% of organic dye within 180 min. Furthermore, the hydrogel with the embedded catalyst exhibits the potential for reusability with a regeneration efficiency of 80.01% after five runs. These findings suggest that this novel hydrogel is a promising candidate for water pollution remediation.

## Introduction

Typically, most of the photocatalysts used in wastewater treatment exist in powder form and are mixed directly into the wastewater stream to form a solid–liquid suspension^1,2^. The degradation of the pollutants takes place under the influence of light. At the end of the pollutant degradation, the photocatalyst powder must be separated and retained for later use. This is a rather difficult problem as nanoscale photocatalysts have low density, agglomerate easily in water and are not suitable for recovery^3^. Although this problem is usually solved by passing the slurry through filter systems, it leads to a loss of photocatalysts which affects the regeneration efficiency of the cycle^4^. Furthermore, it increases the experimental costs when working with expensive photocatalysts. This issue has been addressed by immobilizing the photocatalyst particles on supporting materials, such as activated carbon, cellulose membranes, polymers, etc.^5–9^. Compared with other supporting materials, the conformation of the hydrogel can be flexibly controlled. The adsorption and treatment capacity of catalytic hydrogels compared to other water treatment technologies are faster, lower cost, easier operation, and high efficiency without the generation of by-products. These advantages make it an excellent candidate for use as a substrate in the immobilization of nanoparticle photocatalysts for organic pigment degradation applications. Contributing to promoting the application potential of hydrogels in wastewater treatment, this study develops a method to synthesize hydrogels based on cellulose immobilizing TiO_2_ catalysts with high cellulose content under ambient conditions.

One of the greatest biodegradable and renewable polymers, cellulose, has been studied as a raw material for a newly and fully bio-based hydrogel^10^. However, cellulose presents one of the most insoluble substances due to the intermolecular and intramolecular hydrogen bonds^11^. As a result, choosing an appropriate solution to dissolve cellulose is a challenging initial step in cellulose research. The processes used today involve dissolving cellulose with different solvents such as LiCl/dimethylacetamide^12^, alkali/urea or alkali/thiourea^13^, and ionic liquid solutions^14,15^. These solutions have drawbacks, such as the requirement of high temperatures over 100 ºC or deep cooling at –20 ºC, the use of hazardous chemicals, strict requirements for moisture and difficulty in removing the solvent. Recently, tetrabutylphosphonium hydroxide (TBPH) has become a favorable solvent for dissolving cellulose with advantageous characteristics such as the ability to dissolve a large amount of cellulose (up to approximately 20 *wt*.%) at room temperature in the presence of 50 *wt.*% water^16,17^. In this study, TBPH was used as a solvent to dissolve microcrystalline cellulose so that a large amount of cellulose was dissolved, which was different from other studies in which only hydrogels with low cellulose content were synthesized. With a high cellulose content, the hydrogel material was more stable leading to its ability to be reused many times in the treatment of organic dyes. Meanwhile, there was a better view of the swelling ability, physical and thermal properties of hydrogels with high cellulose content.

Besides, crosslinking is an important step for synthesizing stable hydrogels through two main methods: chemical crosslinking and physical crosslinking^18^. Physical crosslinking involves the entangling of polymer chains, hydrogen bonding, hydrophilic interaction and aggregation. Although not permanent, these physical crosslinks are sufficient to stop hydrogel from dissolving in water. Physically crosslinked hydrogels can absorb water, but the network structure may not be uniform or have defects^19^. Chemical crosslinking could be formed from addition reactions, condensation reactions, ionic bonds, etc., making the hydrogel more resistant and stable^20^. In this study, epichlorohydrin (ECH) is a suitable agent for forming a stable crosslinked network in the hydrogel through substitution reactions between -Cl, epoxide and -OH groups on cellulose chains. ECH, known for its rapid reactivity, chemical stability, and hydrophilicity compared to other crosslinkers, improves the thermal stability and swelling capacity of the hydrogel^21^. Despite being moderately toxic, ECH's toxicity can be reduced after crosslinking^21^. Several studies have demonstrated that ECH is safe for producing biocompatible or biodegradable cellulose hydrogels, offering economic efficiency due to its cost-effectiveness compared to other substances^22,23^.

In addition, titanium dioxide (TiO_2_) was chosen, which represents a type of catalyst that can be incorporated into the hydrogel due to its strong photocatalytic capabilities, chemical stability, and low cost. It is one of the most popular semiconductors photocatalysts in the field of water degradation photocatalysts even it works only under UV irradiation^24–26^.

In this study, we successfully synthesized an “dip-catalyst” hydrogels for the removal of methylene blue (MB) dye, in which the catalyst is immobilized and uniformly distributed within the structure of the hydrogel. This process is more advantageous than other processes due to its simplicity of preparation. The one-pot preparation of hydrogels without catalyst involves the dissolution of microcrystalline cellulose in TBPH 50 *wt.*% and its subsequent crosslinking with ECH. The hydrogel with TiO_2_ samples were obtained using the same method and amount of chemicals, except that a certain amount of TiO_2_ powder was added before the addition of ECH. The new synthesized hydrogel composite material can absorb water contaminants, while the TiO_2_ component can degrade them by photocatalysis. Therefore, the characterization, properties, and morphology of hydrogels have been carefully evaluated, paving the way for applications of hydrogels in the environmental field. The transparency of the hydrogel and its ability to degrade organic dye such as MB have been carefully studied. Moreover, after complete adsorption and treatment of organic dye, the hydrogel was washed, reconstituted, and reused to evaluate the cyclic regeneration efficiency of this material.

## Results and discussion

**Figure 1:**
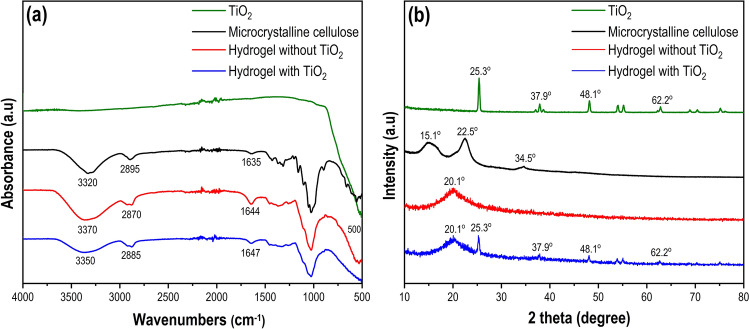
The characterization of microcrystalline cellulose, hydrogel without TiO_2_ (C10E1 sample), hydrogel with TiO_2_ (C10E1Ti10 sample), and TiO_2_ (**a**) FTIR spectra. (**b**) XRD patterns.

Detailed FTIR spectra and XRD analysis were conducted to elucidate the formation of catalytic hydrogel (Fig. 1).

In Fig. 1a, the observed peaks within the wavenumber range of 3500–2800 cm^−1^ in both microcrystalline cellulose and hydrogel were characteristic of stretching vibrations of O–H and C–H bonds^27^. Specifically, the broadband at 3320 cm^−1^ represented the stretching vibration of the hydroxyl groups, encompassing both intra- and intermolecular hydrogen bond vibrations^27^. Notably, the absorption band at 2895 cm^−1^ in the microcrystalline cellulose spectra was associated with C–H symmetric stretching, which shifted to 2870 cm^−1^ in the hydrogel spectrum, indicating successful crosslinking of the cellulose's hydroxyl group^28^. The peak observed at 1635 cm^−1^ for the microcrystalline cellulose shifted to a higher wavenumber at 1647 cm^−1^ and was more intense in the hydrogel spectrum, indicating the interaction between the microcrystalline cellulose and ECH^29^. Therefore, it could be concluded that the observed peak in the hydrogel spectrum was shifted due to the interaction between microcrystalline cellulose and ECH. Besides, the peak at 510 cm^−1^ in the TiO_2_ spectra corresponded to the asymmetric O–Ti–O stretching mode of vibration^30^. Due to the small change, it was assumed that TiO_2_ nanoparticles have no effect on the FTIR spectrum of the hydrogel with TiO_2_. On the other hand, the received signals in the 510 cm^−1^ wavelength range were not very clear, detailed XRD analysis was performed (Fig. 1b). The measurements indicating a crystal transformation in the hydrogels from cellulose I (reflections at 2θ = 15.1º, 22.5º, and 34.5º) to cellulose II (reflections at 2θ = 20.1º)^28^. It can be found that the catalytic hydrogel exhibited the anatase phase of TiO_2_.

**Figure 2:**
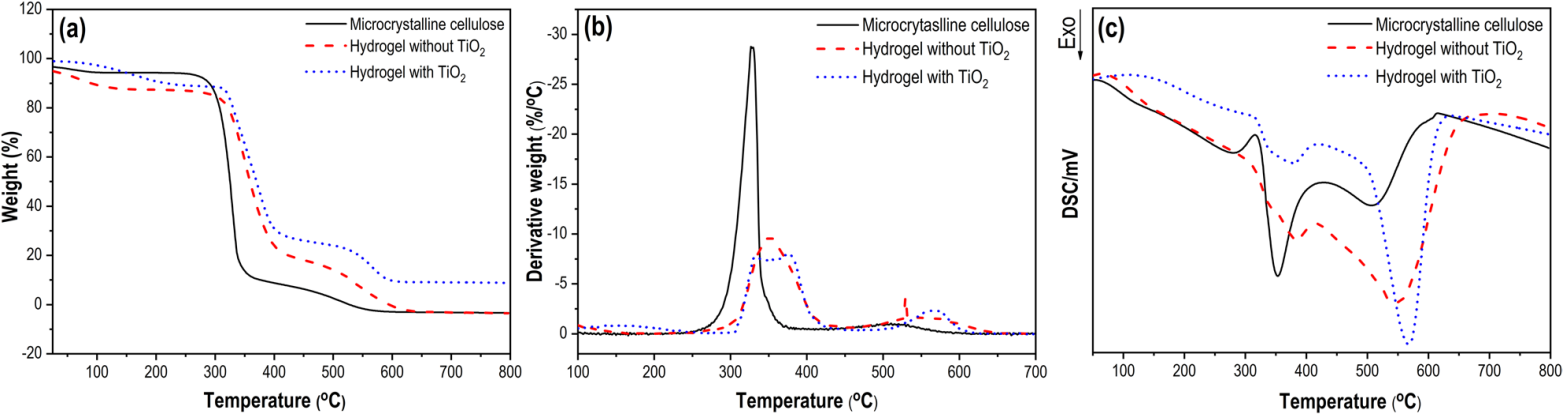
The thermal degradation of microcrystalline cellulose, the hydrogel without TiO_2_ (C10E1 sample) and hydrogel with TiO_2_ (C10E1Ti15 sample) under conditions: air flow rate 50 ml min^−1^, heating rate of 10 °C min^−1^ (**a**) TGA. (**b**) DTG. (**c**) DSC.

TGA, DTG and DSC analyses were performed to investigate the thermal decomposition behavior of microcrystalline cellulose, the hydrogel without TiO_2_, and the catalytic hydrogel sample in a temperature range of 25 ºC to 800 ºC under the air condition (air flow of 50 ml min^−1^) and a heating rate of 10 ºC min^−1^, as shown in Fig. 2. The TGA analysis (Fig. 2a) showed that the thermal decomposition of these samples can be divided mainly into two degradation stages: the slow pyrolysis and the fast pyrolysis. In the temperature range of 25 ºC to 260 ºC, slow pyrolysis takes place and a slight change in the weight of the samples is observed. It can be caused by water loss due to the evaporation and vaporization of moisture from the samples. The thermograms indicated that microcrystalline cellulose underwent minimal changes in mass (3.27%), whereas the hydrogel samples (without and with TiO_2_) exhibited a larger weight loss (8.58% and 10.05%, respectively) with the same rate in the slow pyrolysis stage. The difference in the weight loss behavior was the reduction of the intermolecular forces and the hydrogen bonding resulting from the hydrogel synthesis. When the temperature was in the range of 260 ºC to 400 ºC, the fast pyrolysis stage happened. The weight loss was attributed to the degradation of cellulose chains via glycosidic bond scission due to the combustion of these samples with the oxygen in the air stream. As a result, the weight of the microcrystalline cellulose and the hydrogel without TiO_2_ sample disappears, while a residue remains at the end of the experiment for the hydrogel with TiO_2_ sample.

Figure 2b corresponding to the first derivative of the weight curves (DTG) shows the rate of weight loss for these samples. These samples exhibited the same rate of weight loss in the slow pyrolysis stage. However, in the fast pyrolysis phase, the microcrystalline cellulose sample showed the highest weight loss rate, while the weight loss rate of the hydrogel samples was compatible with each other. In addition, the temperature (326.8 ºC) at which the maximal weight loss rate of the microcrystalline cellulose sample achieved was lower than that (352.7 ºC) at which the maximal weight loss rate of the hydrogel without TiO_2_ sample reached. These results indicate that the thermal stability of the cellulose-based hydrogels was improved. The reason for the improvement is the formation of crosslinks between cellulose fibers in a three-dimensional network^31^.

The DSC analysis (see Fig. 2c) shows that these polymer samples undergo a sequence of structural relaxation, decomposition and oxidation. During the decomposition process, a mixture of flammable gas emitted by the samples reacts with the oxygen in the air stream. An exothermic effect was observed. Following the decomposition stage is the combustion process between the remaining carbonaceous materials and the oxygen in the air flow, which leads to the next exothermic effect in the DSC curves.

**Figure 3:**
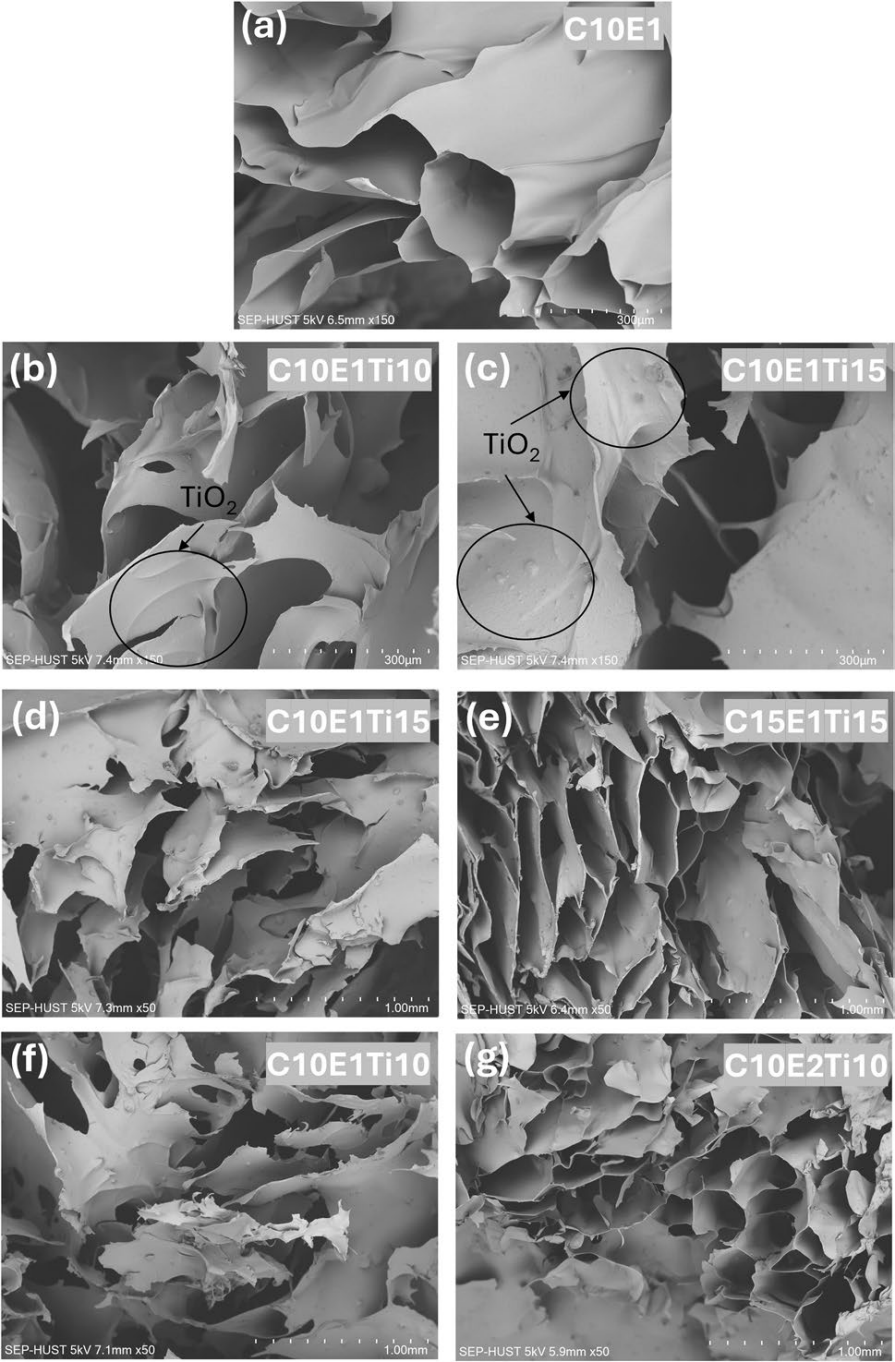
SEM images showing the changes in internal porosity of cellulose hydrogel samples with and without TiO_2_ (**a**–**c**) different amount of catalyst TiO_2_ (without TiO_2_ C10E1, with TiO_2_ C10E1Ti10 and C10E1Ti15, respectively) at 150 × magnification. (**d**, **e**) different composition of microcrystalline cellulose (C10E1Ti15 and C15E1Ti15, respectively) at 50 × magnification. (**f**, **g**) different ECH crosslinker ratio (C10E1Ti10 and C10E2Ti10, respectively) at 50 × magnification.

The hydrogel samples were examined for morphology and microstructure using a scanning electron microscope (SEM) after undergoing freeze-drying (Fig. 3).

The surface morphology and distribution of TiO_2_ in the hydrogel were shown in Fig. 3a, 3b and 3c. In the cross-sectional image, the presence of TiO_2_ particles on the rough surface layers of the C10E1Ti15 and C10E1Ti10 samples were noticeable. Meanwhile, the hydrogel sample without catalyst (C10E1) had a flat and smooth surface layer. The C10E1Ti10 surface layer was smoother than C10E1Ti15 due to the smaller catalytic amount of TiO_2_. Besides, the TiO_2_ particles were observed to be smaller than the hydrogel capillary holes and thus could not be in the pores of the material.

At 50 × magnification, Fig. 3d and 3e showed that the hydrogel consisted of TiO_2_ material has sponge-like network structures. The results illustrated that sample C15E1Ti15 was more porous than sample C10E1Ti15 due to the increase of microcrystalline cellulose content. In addition, the sample C15E1Ti15 has a large number of holes close together, but with smaller sizes than the sample C10E1Ti15. We deduce that as the cellulose concentration increases, the physical crosslinking of the hydrogels becomes stronger due to an increasing number of intermolecular hydrogen bonds and chain entanglements. Consequently, the water permeability of the hydrogels decreases, which in turn affects the material's expansion at room temperature^32^.

The morphology of the hydrogel was affected by the ratio of ECH to OH^−^ as demonstrated by the comparison of the C10E1Ti10 sample with the C10E2Ti10 sample (Fig. 3f and 3g). It can be seen that at the same concentration of cellulose, the pore size of the hydrogel decreases with an increasing ECH:OH^−^ ratio. The C10E1Ti10 has a rather discrete structure with unclear pores, while the C10E2Ti10 sample displayed dense pores with small size. It can be explained that the crosslinking formation in the material due to the reaction of ECH with the -OH group has reduced the pore size but at the same time increased the capillary hole to make the hydrogel structure more stable and sustainable. These results were also completely similar for other hydrogel materials^33^.

**Figure 4:**
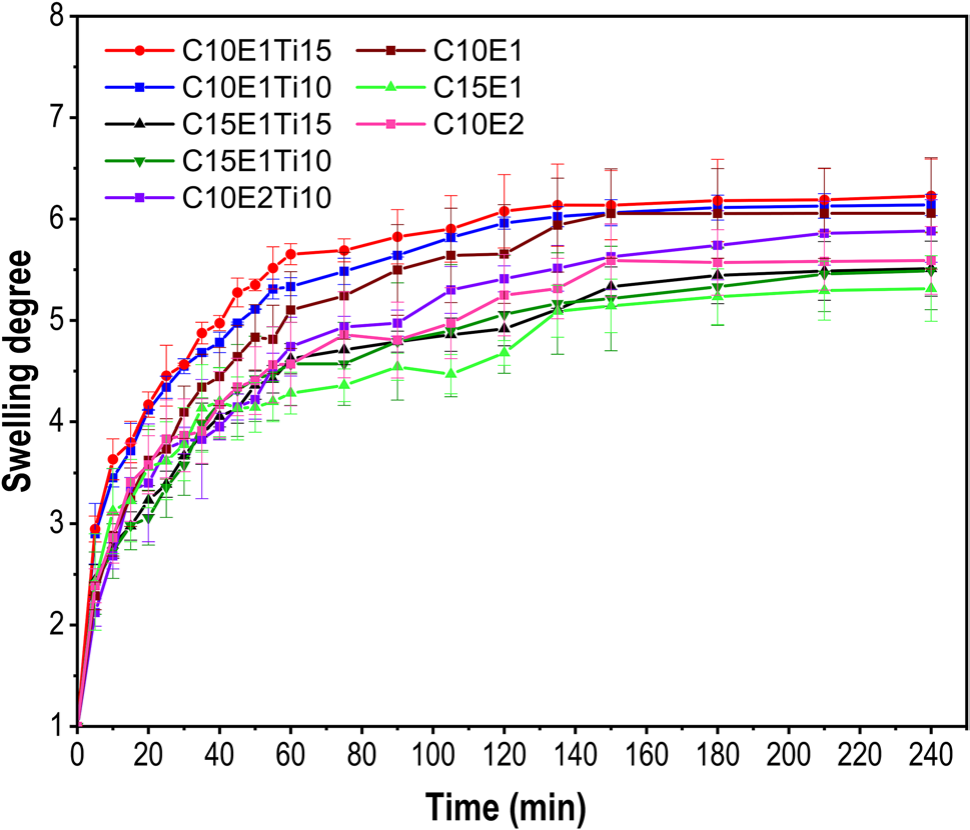
Swelling degree of hydrogels after swelling in 40 ml distilled water for 240 min at 25 ºC as a function of the cellulose concentrations (10 *wt.*% and 15 *wt.*%), the ECH concentrations (ratio 1:1 and 2:1) and amount of TiO_2_ (0 mg, 10 mg and 15 mg).

Figure 4 shows the change in the swelling degree of each hydrogel sample at each specific time when immersed in distilled water at room temperature. All hydrogel samples increased their swelling capacity over time and up to a certain point, they reached swelling equilibrium and had a stable mass. The hydrogel samples expanded very quickly in water in the period from 0 to 60 min. The swelling of the hydrogel occurs due to its increased osmotic pressure, the repulsion between the negative charges promotes swelling by widening the polymer chains. From 60 min onwards, the swelling rate gradually decreased. After about 150 min, the hydrogel samples reached swelling equilibrium. The cross-linked polymers in the hydrogel do not allow solvents or water to dissolve the hydrogel and thus the hydrogel's swelling capacity is limited to a certain extent^34^.

The obtained results align with the crosslink densities of various hydrogel samples calculated according to the Flory-Rehner Eq. (Table 1). When changing the concentration of MCC, the crosslinker ECH amount and the catalyst TiO_2_ content, both the swelling capacity and the crosslink density of the hydrogel also change.

**Table 1:** Correlation between equilibrium swelling degree and crosslink density.

Sample	Mass percent of MCC (*wt.*%)	ECH:OH^-^ ratio	Mass of TiO_2_ per sample (mg)	Maximum of swelling degree (SD)	Crosslink density n (10^−4^ mol cm^−3^)
C10E1	10	1:1	0	6.054 $\pm$ 0.763	1.296 $\pm$ 0.299
C15E1	15	1:1	0	5.144 $\pm$ 0.457	1.756 $\pm$ 0.320
C10E2	10	2:1	0	5.591 $\pm$ 0.625	1.502 $\pm$ 0.296
C10E1Ti10	10	1:1	10	6.138 $\pm$ 0.104	1.226 $\pm$ 0.040
C10E1Ti15	10	1:1	15	6.227 $\pm$ 0.364	1.198 $\pm$ 0.135
C15E1Ti10	15	1:1	10	5.489 $\pm$ 0.383	1.533 $\pm$ 0.208
C15E1Ti15	15	1:1	15	5.510 $\pm$ 0.271	1.517 $\pm$ 0.145
C10E2Ti10	10	2:1	10	5.882 $\pm$ 0.309	1.337 $\pm$ 0.136

C: Microcrystalline cellulose; E: Epichlorohydrin; Ti: Titanium dioxide.

While fixing the content of ECH and TiO_2_ and changing the mass fraction of MCC, the swelling capacity and crosslink density had a certain difference, which was easily recognized in Fig. 4 and Table 1. The higher mass fraction of MCC reduced the swelling degree but raised the crosslink density. In detail, the concentration of MCC was increased by 5 *wt*.%, the swelling degree decreased significantly but the crosslink density increases from 1.226 $\pm$ 0.040 to 1.533 $\pm$ 0.208 for the hydrogel with TiO_2_ (samples C10E1Ti10 and C15E1Ti10); from 1.198 $\pm$ 0.135 to 1.517 $\pm$ 0.145 for samples C10E1Ti15 and C15E1Ti15. The same behavior for the hydrogel without TiO_2_ (samples C10E1 and C15E1) from 1.296 $\pm$ 0.299 to 1.756 $\pm$ 0.320. Additionally, if the hydrogel had a different concentration of ECH crosslinkers, the swelling degree of each sample was also different. The ratio of ECH:OH^‾^ in the hydrogel was adjusted from 1:1 to 2:1, which declined the swelling degree but increased the crosslink density by about 1.091 times (samples C10E1Ti10 and C10E2Ti10). The higher the concentration of the crosslinker was, the lower the swelling degree and the higher the crosslink density was. Furthermore, the variation of the catalyst content can also lead to a change in the swelling capacity and the crosslink density of the hydrogel samples. At swelling equilibrium, hydrogels with TiO_2_ (10 mg or 15 mg) enhanced water absorption due to the increase of hydrophilicity of hydrogel^35,36^. Based on the ANOVA test, the probability value is < 0.05, indicating that the data is significantly different, namely changes in microcrystalline cellulose content had an effect on the swelling value and crosslink density. However, this influence is not significant for the amount of crosslinker and catalyst. Generally, the swelling capacity and crosslink density of hydrogel are inversely related. A higher concentration of the crosslinking agent or cellulose produces the higher crosslink density due to the reaction between ECH and OH^‾^ group. Moreover, the denser crosslinking causes steric effects that limit the interaction of water and the OH^‾^ group in the hydrogel which affects the swelling capacity^33^. Besides, if the catalyst was added to the hydrogel, the TiO_2_ nanoparticles surrounded by –OH groups boosted the hydrophilicity of the hydrogel, making it easier to reach the water molecules^35^.

In order to investigate the photocatalytic degradation efficiency of the hydrogel with TiO_2_ samples, methylene blue (MB) was used as a probe pollutant. MB is classified as a cationic dye that is released as effluents following various industrial processes. Because of its toxicity, MB has to be removed from wastewater.

**Figure 5:**
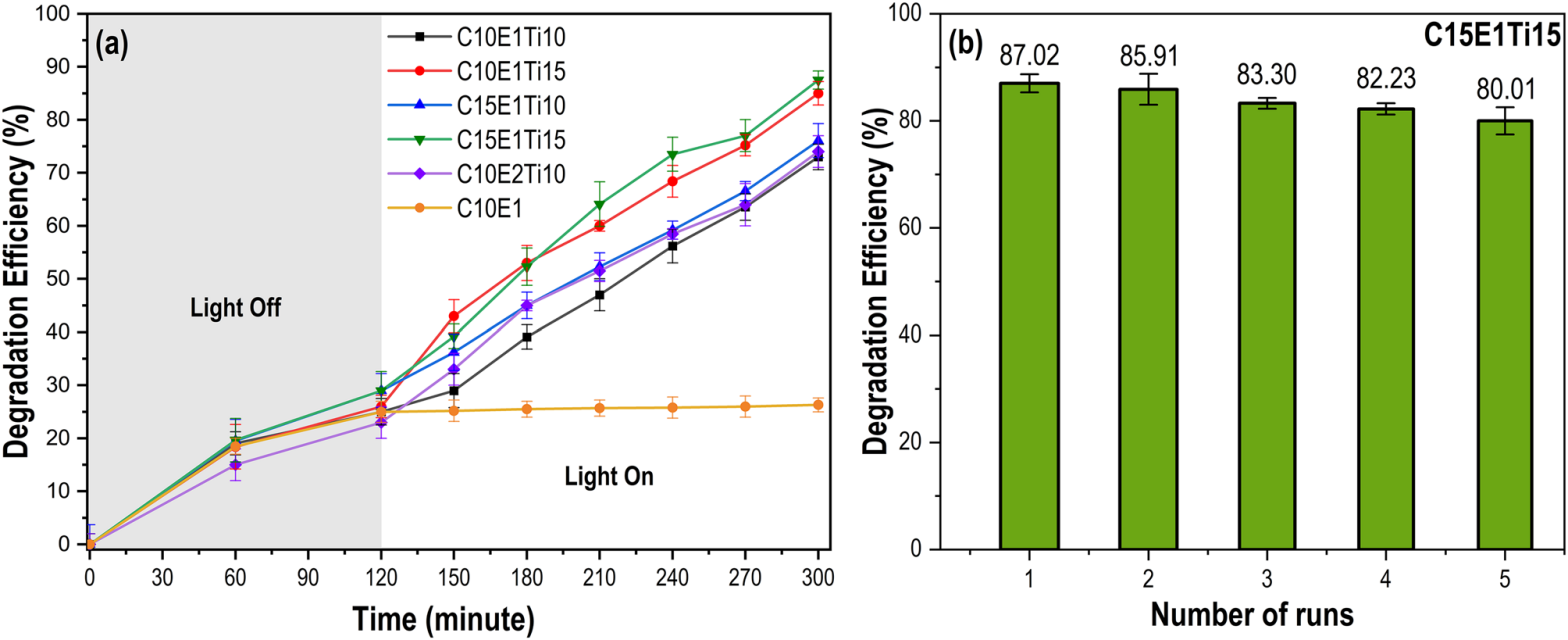
(**a**) Adsorption and photocatalytic degradation efficiency of MB over the different hydrogel without TiO_2_ (C10E1) and hydrogels with TiO_2_ samples. The grey area represents the adsorption of MB dye (120 min, light off). The white area represents photocatalytic degradation efficiency under UV light irradiation (180 min, light on) (**b**) The long-term stability of photocatalytic degradation of hydrogel with TiO_2_ (C15E1Ti15).

Figure 5a shows that the hydrogel samples have photoactivity and can adsorb MB molecules in the absence of light. After 120 min in the dark, the adsorption of MB dye on C10E1, C10E1Ti10, C10E1Ti15 and C10E2Ti10 was negligible, resulting in around 25% MB removal. In contrast, the MB removal was 30% and 32% for C15E1Ti15 and C15E1Ti10 samples, respectively, at equilibrium after the same time. The results indicated that the samples with a cellulose content of 15% (C15E1Ti15 and C15E1Ti10) have a better adsorption performance than the samples with a cellulose content of 10 *wt.*% (C10E1, C10E1Ti10, C10E1Ti15 and C10E2Ti10). This can be explained by the high cellulose content and its negative zeta potential^37^. Microcrystalline cellulose possesses hydrophilic functional groups, such as hydroxyl (-OH), which enable the formation of both physical crosslinking and chemical crosslinking. This creates a 3D structure that enhances the adsorption of MB in a solution, primarily through van der Waals forces and electrostatic interactions. Besides, the adsorption capacity of the hydrogel with TiO_2_ samples was notably influenced by the ECH ratio, as demonstrated by comparing the C10E1Ti10 sample with the C10E2Ti10 sample. It was evident that, under the same adsorption duration, C10E1Ti10 exhibited a higher MB adsorption capacity compared to C10E2Ti10. The presence of ECH in hydrogel synthesis played a vital role in crosslinking polymer chains, thereby influencing the pore structure and overall surface properties of the hydrogel. Higher concentrations of ECH tended to result in a more densely crosslinked hydrogel matrix with reduced porosity. Conversely, lower ECH concentrations may yield hydrogels with a more open and porous structure, potentially leading to increased MB adsorption due to the higher surface area and greater exposure of active sites.

In addition to the adsorption capacity, another factor that played an important role in MB treatment was the photoactivity of the hydrogel with TiO_2_. Under UV light irradiation by the Xenon lamp, the TiO_2_ was excited, and the photogenerated charge carriers could be transferred to the nearby MB and participate in the redox reactions, leading to the decomposition of MB into CO_2_ and H_2_O. By comparing samples with similar amount of ECH and microcrystalline cellulose but different TiO_2_ content, the influence of catalyst on the photocatalytic activity was assessed (p < 0.05). More than 85% of the initial dye were decomposed by C10E1Ti15 and C15E1Ti15 after 180 min. In contrast, nearly 30% of the initial dye remained in the solution after the same period of time for C10E1Ti10, C15E1Ti10 and C10E2Ti10. This showed that the prepared hydrogel with TiO_2_ samples have good photocatalytic activity for MB. As expected, C10E1Ti15 and C15E1Ti15 had the highest MB degradation efficiency because they had the most distributed TiO_2_ content in the hydrogel structure. These created favorable conditions for the formation of numerous active sites within the hydrogel, which enhance light absorption and generate holes and electrons during MB degradation. Moreover, the high transparency of the hydrogel was indeed an advantage for the photocatalytic process^37^. The transparency of the hydrogel enables light to penetrate the material, enhancing the effective interaction of TiO_2_ with photon energy. Thus, the cellulose-based hydrogel supported TiO_2_ catalyst was an efficient strategy for MB remediation due to its combination of adsorption and degradation.

Figure 5b illustrates the evaluation of the reusability of hydrogel with TiO_2_ photocatalytic degradation. Each cycle lasted 180 min and five successive cycles were used to control the photocatalytic breakdown of MB. After each cycle, the hydrogel with TiO_2_ was taken out of the MB solution and thoroughly washed with water, then placed in a fresh MB solution. The elimination efficiency was 87.02% in the first cycle, and for the following four cycles, it dropped 1.087 times in the final cycle (80.01%). The MB removal efficiency under UV light did not decrease significantly during five consecutive cycles using one-way ANOVA test, which proved the excellent regenerative ability and durability of the hydrogel with TiO_2_ against the photodegradation of MB.

## Materials and methods

### Materials

Microcrystalline cellulose (MCC, powder, size 20 μm, CAS No.: 9004–34-6), Titanium dioxide (TiO_2_, CAS No.: 13463–67-7), Epichlorohydrin (ECH, 99%, CAS No.: 106–89-8) and Methylene blue (MB, powder, CAS No.: 122965–43-9) were purchased from Sigma–Aldrich. Tetrabutylphosphonium hydroxide (TBPH 40 *wt.*%, CAS No.: 14518–69-5) was obtained from Acros.

### Methods

#### Fabrication of cellulose hydrogels without TiO_2_

**Figure 6:**
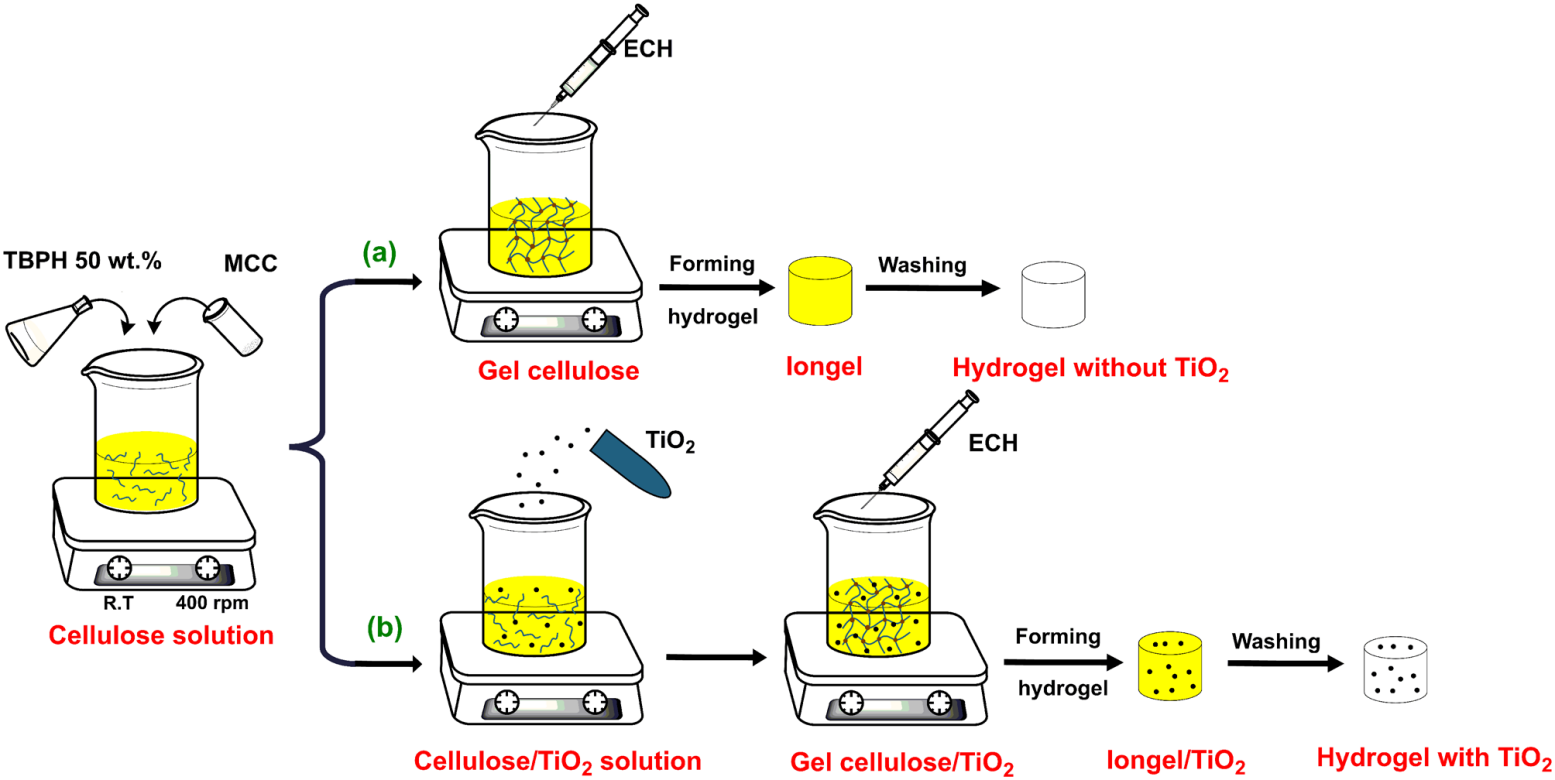
Schematic of the hydrogel materials production (**a**) Cellulose hydrogel without TiO_2_. (**b**) Cellulose hydrogel with TiO_2_.

This preparation of a cellulose-based hydrogel without TiO_2_ is shown in Fig. 6a and Table 2. The process started by dissolving microcrystalline cellulose in 1 ml TBPH 50 *wt.*% for 30 min at 400 rpm at room temperature. The commercial TBPH 40 *wt.*% was condensed to higher concentration (TBPH 50 *wt.*%) by using rotary evaporation at 60 mbar below 40 ºC. Microcrystalline cellulose was dissolved in different concentrations (10 *wt.*% and 15 *wt.*%) to get cellulose solution. Then, the ECH crosslinker was added to the cellulose solution and stirred to obtain a homogeneous gel cellulose solution. The crosslinker ratio between ECH:OH^−^ was 1:1 and 2:1. The gel solution was molded into shape at room temperature (25 ºC) to obtain an iongel. After that, the iongel was washed many times with distilled water for one day to remove the TBPH solvent and obtain a hydrogel (denoted as Hydrogel without TiO_2_).

**Table 2:** Investigation of the synthesis process of hydrogel samples with and without TiO_2_.

Sample	TBPH 50%^a^ (ml)	MCC (*wt.* %)^b^	ECH (ml)	ECH:OH^−^ ratio	Mass of TiO_2_ (mg)	Reaction conditions	
						Temperature (^o^C)	Time (min)
C10E1	1	10	0.1427	1:1	0	RT	10
C10E2	1	10	0.2854	2:1	0	RT	9
C15E1	1	15	0.2141	1:1	0	RT	7
C10E1Ti10	1	10	0.1427	1:1	10	RT	10
C10E1Ti15	1	10	0.1427	1:1	15	RT	10
C15E1Ti10	1	15	0.2141	1:1	10	RT	7
C15E1Ti15	1	15	0.2141	1:1	15	RT	7
C10E2Ti10	1	10	0.2854	2:1	10	RT	9

C: Microcrystalline cellulose; E: Epichlorohydrin; Ti: Titanium dioxide; RT: room temperature (25 °C).

^**a**^Water content: 50%.

^**b**^Compared to TBPH 50 *wt.*% solution.

#### Fabrication of cellulose hydrogels with TiO_2_

The hydrogel with TiO_2_ sample was obtained with the same method and amount of chemicals except for the addition of 10 mg and 15 mg of TiO_2_ powder before adding ECH (Fig. 6b and Table 2).

### Characterization

#### Fourier transform infrared spectroscopy (FT-IR)

Samples were analyzed using an FT-IR spectrometer (Nicolet iS50, Thermo Fisher Scientific) in ATR mode. This equipment uses a monolithic diamond crystal as an ATR material. The measurements were taken with 16 scans in the range from 400 to 4000 cm^−1^ with a resolution of 4 cm^−1^. The measurements were done with of microcrystalline cellulose, hydrogel without TiO_2_ (C10E1 sample), hydrogel with TiO_2_ (C10E1Ti10 sample), and TiO_2_.

#### X-ray diffraction (XRD)

Powder patterns were recorded using X'Pert Pro XRD equipment (PANalytical) under Cu kα radiation (40 kV, 35 mA). The measurements were conducted using microcrystalline cellulose, hydrogel without TiO_2_ (C10E1 sample), hydrogel with TiO_2_ (C10E1Ti10 sample), and TiO_2_.

#### UV–Vis spectroscopy

UV–Vis spectroscopy was used to assess the transparency of solid samples. The light transmission of the substrate and hydrogels was compared to that of distilled water as a reference (baseline). The hydrogel was positioned perpendicular to the light beam in a cuvette. The spectra of the samples were obtained using an Avantes UV–Vis spectrometer.

#### Scanning electronic microscopy (SEM)

The morphologies of the freeze-dried cellulose hydrogels were carried out using a Hitachi Tabletop Microscopes TM 4000 Plus at an accelerating voltage of 5 kV with a 5 nm gold coating (Quorum Q150T S Plus Sputter Coater). Before the observation in SEM, the hydrogel samples were swollen to equilibrium, and then immersed in liquid nitrogen. The frozen samples were lyophilized by the vacuum freeze drier (Labconco Freezone 4.5 Liter Freeze Dry System). In the process of gold coating, the sample is positioned in the sputter coater vacuum chamber where gold is sputtered onto its surface using argon plasma.

#### Thermogravimetric analysis (TGA) and Differential scan calorimeter (DSC)

TGA and DSC were performed on a single instrument on the STA 449 F1 Simultaneous TGA/DSC from Netzsch GmbH Instruments. The tests were conducted with aluminum crucibles under air atmosphere with a temperature range of 25 ºC to 800 ºC and a heating rate of 10 ºC min^−1^. All the data was recorded and analyzed using Proteus Software on Windows. The measurements were done with microcrystalline cellulose, hydrogel without TiO_2_ (C10E1 sample), hydrogel with TiO_2_ (C10E1Ti10 sample), and TiO_2_.

#### Evaluation of swelling behavior

The swelling behavior of the hydrogel was tested as a function of cellulose concentration, the ratio of crosslinker and the amount of catalyst. The swelling capacity of the hydrogel was evaluated by immersing the aerogel samples in distilled water at room temperature (25 °C) within a certain time period. The complete swelling degree was characterized by the maximum amount of adsorbed water, which was determined when the mass of the swollen hydrogel remained unchanged. To evaluate the swelling capacity of the hydrogel, the swelling degree (SD) was calculated by Eq. 1^38^:

1 $$SD = \frac{{m_{s} }}{{m_{d} }}$$

where m_s_ is the mass of swollen hydrogel at a certain time (gram), m_d_ is the mass of the absolute dry hydrogel (gram).

The swelling degree of each hydrogel sample was calculated as the average of the results of two measurements.

#### Evaluation of crosslinker density

The formation of cross-links between the cellulose molecule and the ECH crosslinker leads to different degrees of swelling of the hydrogel samples. The equilibrium swelling of the hydrogel in terms of crosslinking density and solvent strength will be described by the Flory-Rehner equation (Eq. 2)^39^. This equation is written as:

2 $$n = - \frac{{\ln \left( {1 - V_{p} } \right) + V_{p} + \chi V_{p}^{2} }}{{V_{0} \left( {V_{p}^{\frac{1}{3}} - \frac{{V_{p} }}{2}} \right)}}$$

where n is the crosslink density (mol cm^−3^), V_p_ is the volume fraction of the polymer in the swollen polymer, χ = 0.44 is the Huggins hydrogel-water interaction constant, V_0_ = 18 is the molar volume of water (cm^3^ mol^−1^).

Specifically, V_p_ is calculated according to Eq. 3^40^:

3 $$V_{p} = \frac{{\frac{1}{{D_{p} }}}}{{\frac{SD}{{D_{0} }} + \frac{1}{{D_{p} }}}}$$

where D_0_ = 0.997 is the density of water (g cm^−3^), D_p_ = 1.5 is the density of cellulose (g cm^−3^), SD is the swelling degree calculated based on Eq. 1.

#### Photocatalytic activity evaluation

The photocatalytic activity of the hydrogel with TiO_2_ was evaluated by assessing its ability to degrade methylene blue (MB) as a model pollutant in an aqueous solution under ambient temperature conditions. Specifically, cylindrical hydrogel samples measuring approximately 100 × 80 mm were immersed in a 50 ml solution containing 10 ppm of MB. The pH of the MB solution is 6.76. The samples were then placed in a dark environment for a period of 120 min to allow for the establishment of adsorption–desorption equilibrium. The hydrogel with TiO_2_ containing solution underwent 180 min of UV irradiation using a 300W Xenon lamp. At regular 30 min intervals during irradiation, samples were collected to assess the photocatalytic activity. The concentration of MB was determined by measuring its absorbance at a wavelength of 664 nm using a UV–Vis spectrophotometer. Subsequently, the degradation efficiency was computed, and the degradation percentages of MB in the aqueous solution were calculated using the following formula (Eq. 4):

4 $$\% Degradation = \frac{{C_{0} - C_{t} }}{{C_{0} }} \cdot 100\%$$

where C_o_ represents the dye solution concentration at adsorption equilibrium, while C_t_ signifies the solution concentration after time t.

### Statistical analysis

All quantitative data were obtained in triplicate and were expressed as mean ± standard deviation (SD). Significant differences between experimental groups were determined by two-way analysis of variance (ANOVA) for swelling degree, crosslink density and photoactivity measurement. One-way ANOVA was used to compare the effect of one variable for the reusability test of sample C15E1Ti15. p value < 0.05 was considered significant. Data analysis was performed using Excel Microsoft Office 365 (Microsoft Corporation, Inc., Washington, United States).

## Conclusions

An environmentally friendly cellulose-based hydrogel with photocatalyst has been successfully fabricated via a simple, and one-step method. TBPH, a superior solvent compared to others, has been used to dissolve microcrystalline cellulose. Therefore, a large amount of cellulose for hydrogel synthesis can be dissolved (10 *wt.*% and 15 *wt.*%) resulting in a better view of the swelling ability and their properties. This has important implications for the use of photocatalytic hydrogels as the photocatalyst and adsorbent for the removal of MB. The characterization, morphology, and thermal stability of these samples were investigated carefully. The hydrogel with TiO_2_ has porous structures with the distribution of TiO_2_ in the layer of hydrogel. The swelling capacity of the hydrogel was inversely proportional to its crosslinking density, which was evaluated with different contents of microcrystalline cellulose, ECH, and TiO_2_. High crosslinking density leads to a more rigid and stable hydrogel. The photocatalytic activity of the samples was demonstrated through the excellent degradation of organic dye that successfully removed MB (≈90%). In addition, this was also a suitable material for reuse due to its high regeneration efficiency. Therefore, they exhibited outstanding properties that make them suitable stable supports for immobilizing photocatalysts as well as excellent adsorbents for the removal of organic dye.

## Data Availability

All data generated or analysed during this study are included in this published article.
